# Identification of key interferon-stimulated genes for indicating the condition of patients with systemic lupus erythematosus

**DOI:** 10.3389/fimmu.2022.962393

**Published:** 2022-07-28

**Authors:** Mengjia Shen, Congcong Duan, Changhao Xie, Hongtao Wang, Zhijun Li, Baiqing Li, Tao Wang

**Affiliations:** ^1^ Department of Rheumatology and Clinical Immunology, The First Affiliated Hospital of Bengbu Medical College, Bengbu, China; ^2^ Anhui Provincial Key Laboratory of Immunology in Chronic Diseases, Bengbu Medical College, Bengbu, China

**Keywords:** systemic lupus erythematosus, interferon, interferon-stimulated gene, bioinformatics, GEO database, long non-coding RNA

## Abstract

Systemic lupus erythematosus (SLE) is a chronic autoimmune disease with highly heterogeneous clinical symptoms and severity. There is complex pathogenesis of SLE, one of which is IFNs overproduction and downstream IFN-stimulated genes (ISGs) upregulation. Identifying the key ISGs differentially expressed in peripheral blood mononuclear cells (PBMCs) of patients with SLE and healthy people could help to further understand the role of the IFN pathway in SLE and discover potential diagnostic biomarkers.

The differentially expressed ISGs (DEISG) in PBMCs of SLE patients and healthy persons were screened from two datasets of the Gene Expression Omnibus (GEO) database. A total of 67 DEISGs, including 6 long noncoding RNAs (lncRNAs) and 61 messenger RNAs (mRNAs) were identified by the “DESeq2” R package. According to Gene Ontology (GO) enrichment analysis and Kyoto Encyclopedia of Genes and Genomes (KEGG) pathway enrichment analysis, those DEISGs were mainly concentrated in the response to virus and immune system processes. Protein-protein interaction (PPI) network showed that most of these DEISGs could interact strongly with each other. Then, IFIT1, RSAD2, IFIT3, USP18, ISG15, OASL, MX1, OAS2, OAS3, and IFI44 were considered to be hub ISGs in SLE by “MCODE” and “Cytohubba” plugins of Cytoscape, Moreover, the results of expression correlation suggested that 3 lncRNAs (NRIR, FAM225A, and LY6E-DT) were closely related to the IFN pathway.

The lncRNA NRIR and mRNAs (RSAD2, USP18, IFI44, and ISG15) were selected as candidate ISGs for verification. RT-qPCR results showed that PBMCs from SLE patients had substantially higher expression levels of 5 ISGs compared to healthy controls (HCs). Additionally, statistical analyses revealed that the expression levels of these ISGs were strongly associated to various clinical symptoms, including thrombocytopenia and facial erythema, as well as laboratory indications, including the white blood cell (WBC) count and levels of autoantibodies. The Receiver Operating Characteristic (ROC) curve demonstrated that the IFI44, USP18, RSAD2, and IFN score had good diagnostic capabilities of SLE.

According to our study, SLE was associated with ISGs including NRIR, RSAD2, USP18, IFI44, and ISG15, which may contribute to the future diagnosis and new personalized targeted therapies.

## Introduction

SLE is a typical chronic autoimmune disease that primarily affects women of childbearing age. Patients with SLE may suffer symptoms such as fever, arthritis, rashes, serositis, cytopenias, renal disease, and other symptoms (1). Patients with SLE range in severity, with the most severe instances being potentially fatal. The heterogeneity of clinical manifestations among patients may be caused by the diversity of potential molecular mechanisms (2).

Although the precise molecular mechanisms are not entirely apparent, it is currently thought that the pathogenesis of SLE is linked to immunological regulation, inheritance, environmental variables, sex, epigenetics, and other factors (1). Several investigations have demonstrated that type I IFN (IFN-I), particularly IFN-α, is the core pathogenic mediator in SLE (3). IFN-I triggers abound in SLE patients and include immune complexes containing host nucleic acids. The plasmacytoid dendritic cell (pDC) is the major cell type for IFN-I production. There is extensive crosstalk going on between other immune cells and pDC to jointly promote the continuous production of IFN-I at the same time (4–7). In addition, a variety of factors, including African ancestry, UV light, infection, medications, and estrogen, have been reported to be inducers of IFN production (8–11). IFN-I exerts impacts on innate and acquired immunity in SLE aside from its direct antiviral properties. These effects include boosting immune cells proliferation, differentiation, maturation, or death, and encouraging B lymphocytes to produce autoantibodies (5, 11). Persistent production of IFN-I can cause aberrant autoimmune responses and chronic inflammation, eventually resulting in tissue damage (6, 12, 13).

At the same time, a substantial amount of IFN-I binds to the receptors IFNAR1 and IFNAR2, triggering a signal cascade through some pathways, including the most well-known JAK/STAT pathway, resulting in ISGs overexpression in blood and tissue, a phenomenon named “IFN signature”. High level of type I IFN signature is also considered to be one of the driving forces of SLE progression (5, 14, 15). Upregulation of type I ISGs’ expression was identified in 60% to 80% of SLE patients, and those with high IFN signature tended to show higher disease activity and autoantibodies levels (16).

Glucocorticoid (GC) and hydroxychloroquine (HCQ), which can temporarily ease symptoms are the mainstays of SLE treatment today. Future treatment may concentrate on tailored treatment based on molecular processes as SLE is the outcome of various etiology, appearing differently in terms of manifestations, therapeutic responses, and prognosis (9). Monoclonal antibodies against IFN-I signaling have shown a considerable effect in recent years, and Anifrolumab monoclonal antibody is one of them. Anifrolumab has been demonstrated in clinical trials to exhibit consistent efficacy and safety in patients with mild to severe SLE because it inhibits IFN-I signaling by binding to IFN-I receptors (17–20). Drugs like Anifrolumab are largely dependent on the presence of IFN signature, which is utilized to stratify patients in relevant clinical trials generally. As a result, the IFN signature is expected to become a more conventional parameter for diagnosing SLE in the future not only for signaling the activation degree of the IFN-I pathway (21, 22). However, there is no consistent ideal standard of ISGs for quantifying IFN signature, and the pathogenic mechanisms of those ISGs associated with SLE are not yet fully understood. As a result, this study aims to identify significant ISGs which may play key roles in occurrence and progression of SLE.

## Materials and methods

### Datasets collection

The Gene Expression Omnibus (GEO) database^1^ was used to obtain two datasets, one of which was used to identify differentially expressed genes (DEGs) in PBMCs of SLE patients and healthy controls. So the SLE disease group and healthy control group should be included in the sample groupings, and there should be at least 3 samples in each group. Another dataset for identifying possible type I ISGs took “sample groups including IFN-α treated group and IFN-α untreated group with the number of samples in each group≥3” as the condition. Human PBMCs serve as the sample source in both datasets. The GSE122459 and GSE159094 datasets were chosen as the target data source of this study. The **Supplementary Table 1** contains detailed information of two datasets.

### Screening of differentially expressed interferon-stimulated genes

DEGs were identified from the raw data of two datasets by the “DESeq2” R package in R software (Version 4.1.0) (23) with the threshold “|log_2_FC|≥1 and *p*-value < 0.05”. The genes without ensemble gene ID were removed. Next, the results were visualized as volcano plots through the Sangerbox platform^2^ (24). Then, A Venn diagram was created by an online tool^3^ to confirm the overlapping DEGs of the two datasets. We regarded those overlapped DEGs as potential differentially expressed type I ISGs in PBMCs between SLE patients and healthy controls. The heatmaps of DEGs and DEISGs in GSE122459 were created by another online tool^4^.

### Functional annotation and pathway enrichment analysis

Gene Ontology (GO) analysis annotates DEGs in terms of biological processes (BP), cellular compositions (CC), and molecular functions (MF). Kyoto Encyclopedia of Genes and Genomes (KEGG) analysis is regularly used to predict the potential biological function of DEGs. In order to better comprehend the potential function of DEISGs, we examined the enrichment of GO and KEGG through the GeneCodis website^5^ and visualize the results through the Sangerbox platform.

### Protein-protein interaction network construction and hub ISGs identification

The Search Tool for the Retrieval of Interacting Genes/Proteins (STRING) website^6^ was used to construct a PPI network for the protein-coding genes in DEISGs with the cutoff interaction score set at 0.4. The network was then visualized using Cytoscape software (version 3.9.0). Next, the “MCODE” plugin of Cytoscape was performed with default parameters to discover the most important clustering module in the PPI network. Another plugin “Cytohubba” was used to assign values to each gene through the topological network algorithm, and the top 10 genes with the highest maximal clique centrality (MCC) values based on centrality, eccentricity, and radiality, were selected as the hub genes in the PPI network.

### Collection of patients and healthy controls

This study comprised a total of 58 SLE patients and 38 healthy controls. All SLE patients were recruited from the Department of Rheumatology and Clinical Immunology at the First Affiliated Hospital of Bengbu Medical College, and they all met the 1997 American College of Rheumatology (ACR) SLE criteria (25). Patients with other autoimmune disorders, severe infections, or malignant conditions were excluded. The disease activity was determined by the SLE disease activity index 2000 (SLEDAI-2K) (26). Meanwhile, clinical information such as clinical symptoms and laboratory indicators were also collected. At the same period, age- and gender-matched healthy individuals from the Health Examination Center were recruited as controls. The basic characteristics of the SLE group and HC group are shown in **Table 1**. This study was carried out in accordance with the principles of the Helsinki Declaration and approved by the Human Ethics Committee of the First Affiliated Hospital of Bengbu Medical College.

**Table 1:** General information characteristics of SLE patients and healthy controls.

Group	Sex (male/female)	Age (years)	Disease duration (years)	SLEDAI-2K	HCQ applications (yes/no)	GC applications (yes/no)
HC	7/31 ^(1)^	38.82 (21-64) ^(1)^	/	/	/	/
SLE	7/51	39.50 (12-74)	4.92 (0-20)	8.21 (0-23)	44/14	53/5
LN	3/23^(2)^	41.35 (18-66)^(2)^	6.11 (0.25-20)^(3)^	10.85 (0-23)^(4)^	23/3^(3)^	25/1 ^(2)^
NLN	4/28	38.00 (12-74)	3.96 (0-12)	6.06 (0-14)	21/11	28/4

Data are shown as the N or Mean (Range). HC: Healthy controls; LN: SLE patients with lupus nephritis; NLN: Non-lupus nephritis SLE patients; (1) Compared with other three groups, p > 0.05; (2) Compared with NLN group, p > 0.05; (3) Compared with NLN group, p < 0.05; (4) Compared with NLN group, p < 0.001.

### Total RNA extraction and RT-qPCR

Fasting peripheral blood (5ml) from patients and healthy controls were collected by ethylene diamine tetraacetic acid (EDTA) tubes in the morning. PBMCs were isolated and extracted using the Ficoll gradient centrifugation protocol after centrifugation separating plasma. Total RNA was extracted by chloroform and TRIzol Reagent (Invitrogen, CA, USA). EasyScript^®^ One-Step gDNA Removal and cDNA Synthesis SuperMix (Transgen, Beijing, China) was used to reverse transcribe cDNA. The RT-qPCR reaction was carried out in the LightCycler^®^ 96 real-time PCR System (Roche Diagnostics GmbH, Mannheim, Germany) using 2 × Perfect Start genes Green qPCR Supermix (Transgen, Beijing, China) according to the instructions. The amplification curve, melting curve, and Cq value were analyzed. The Cq values of the target genes and the internal control gene β-actin were used to calculate the relative expression of every target gene in each sample by the 2^−△△Cq^ method. The primer sequences of 5 ISGs and β-actin were listed in **Supplementary Table 2**.

### Calculation of the IFN Score

Since there is no consensus on which ISGs should be utilized to construct the IFN score, this study selected NRIR, RSAD2, USP18, IFI44, and ISG15 for calculation according to the algorithm introduced in the literature (27). Briefly, the expression level of each ISG in the HC group served as a criterion for the expression level of each ISG in each subject. The IFN score was an overall score calculated by summing the standardized expression level of each ISG per subject.

### Statistical analysis

Data were analyzed by using GraphPad Prism (Version 9.0, GraphPad Software, CA, USA) and SPSS (Version 26, IBM, New York, USA). The data in this study mostly did not satisfy normal distribution, so the median and interquartile range (IQR) were mainly used for data description unless specified. The nonparametric Mann-Whitney U test was performed to compare every ISG expression between the two groups. The counting data were expressed by frequency, and the chi-square test was performed to compare the differences between the two groups. The correlation was analyzed by Spearman’s test. ROC analysis was used to evaluate the diagnostic efficacy of hub ISGs and IFN scores for differentiating SLE patients from healthy controls. Two-tailed *p*-value < 0.05 was deemed statistically significant.

## Results

### Differentially expressed ISGs between the SLE patients and healthy controls samples and functional analysis

**Figure 1:**
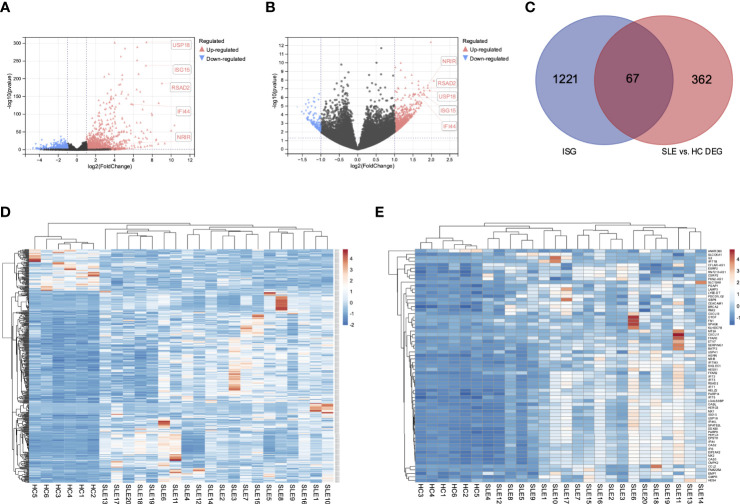
Differential expressed interferon-stimulated genes in SLE patients and healthy controls. **(A)** Volcano of the DEGs of GSE159094. **(B)** Volcano of the DEGs of GSE122459. **(C)** Venn diagram of the overlapped DEGs of two datasets. **(D)** Heatmap of the 429 DEGs in SLE patients and healthy controls. **(E)** Heatmap of the 67 DEISGs in SLE patients and healthy controls.

We obtained a total of 1288 genes, including 105 lncRNAs and 1183 mRNAs, up-regulated in PBMCs after IFN-α stimulation (**Figure 1A**). These genes were considered potential type I ISGs. Then we screened a total of 429 DEGs (including lncRNAs and mRNAs) in PBMCs of SLE patients and healthy controls (**Figure 1B**), of which 352 DEGs were up-regulated and 77 DEGs were down-regulated. Venn diagram identified 67 overlapped genes (**Figure 1C**), including 66 up-regulated genes (6 lncRNAs and 60 mRNAs) and 1 down-regulated mRNA. These genes could be type I ISGs expressed differentially in PBMCs of SLE patients and healthy people. The heatmaps of DEGs and DEISGs are presented in **Figures 1D**, **E**. The top 10 ISGs with the biggest multiple changes between SLE patients and HCs are listed in **Table 2**.

**Table 2:** The Top 10 ISGs that were significantly and differentially expressed in PBMCs of SLE patients and healthy controls.

Gene name	Log_2_FC	*P* value	Up/down regulated	Gene type
**IFI44L**	2.067	1.12402E-08	Up	mRNA
**FFAR2**	1.983	3.86476E-13	Up	mRNA
**NRIR**	1.903	3.82729E-08	Up	lncRNA
**RSAD2**	1.901	7.35401E-08	Up	mRNA
**USP18**	1.799	1.5302E-07	Up	mRNA
**IFIT3**	1.790	3.24493E-07	Up	mRNA
**CMPK2**	1.709	2.43784E-07	Up	mRNA
**ISG15**	1.698	2.35767E-07	Up	mRNA
**IFI6**	1.671	5.80257E-07	Up	mRNA
**IFIT1**	1.657	5.72341E-06	Up	mRNA

**Figure 2:**
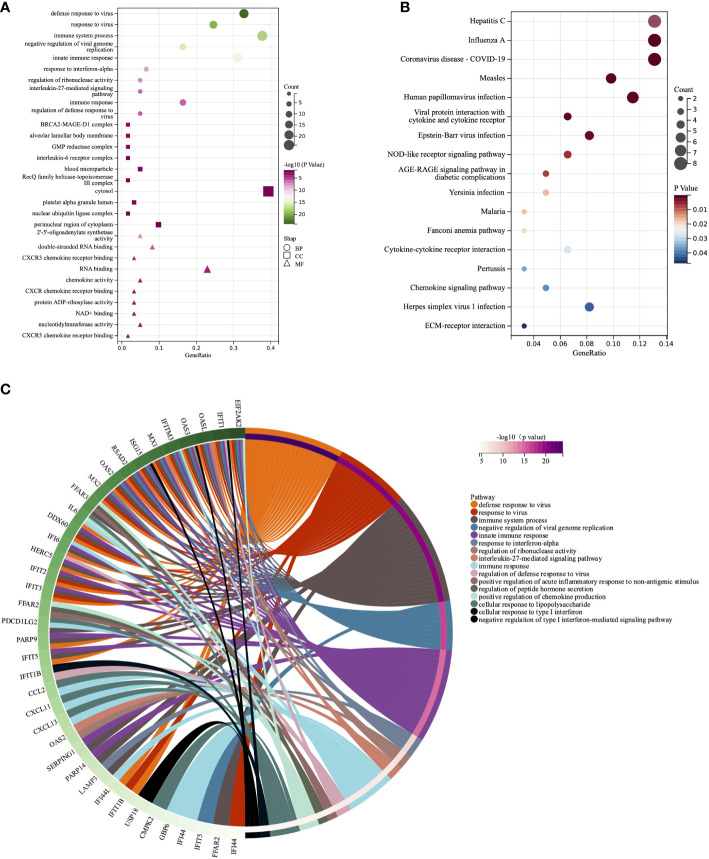
GO and KEGG analyses of DEISGs. **(A)** Biological process, cell components, and molecular function enrichment analyses of DEISGs. **(B)** The KEGG analyses of DEISGs. **(C)** Distribution of DEISGs for biological process enrichment.

To further investigate the potential functions of these genes, we carried out GO and KEGG enrichment analyses. The findings showed that the biological processes of DEISGs were predominantly focused on defense response to virus, immune system process, and response to interferon-alpha (**Figures 2A**, **C** **)**. BRCA2-MAGE-D1 complex, blood microparticle, alveolar lamellar body membrane, and interleukin-6 receptor complex were the main related cellular components (**Figure 2A**). The molecular functions mainly enriched were 2’-5’-oligoadenylate synthetase activity, RNA binding, chemokine activity, and CXCR chemokine receptor binding (**Figure 2A**). Furthermore, the enrichment of KEGG revealed that DEISGs were mainly involved in virus-related diseases such as Hepatitis C, Influenza A, and COVID-19, viral protein interaction with cytokine and cytokine receptor, and NOD-like receptor signaling pathway (**Figure 2B**).

### Identification of hub ISGs

**Figure 3:**
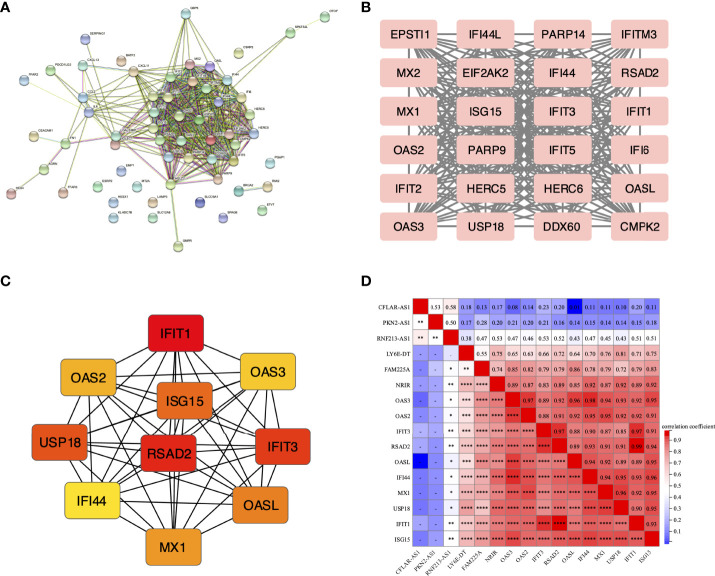
Interactions among the DEISGs. **(A)** The PPI network in DEISGs. **(B)** The most significant module in the PPI network. **(C)** Top 10 hub ISGs based on maximal clique centrality (MCC). **(D)** The correlation heatmap of the 6 lncRNAs and 10 hub ISGs. *P < 0.05, **P < 0.01, ***P < 0.001, ****P < 0.0001, -*P* ≥ 0.05

The protein-protein interaction was obtained from the STRING database (**Figure 3A**). The most important clustering module in DEISGs was obtained through the “MCODE” (**Figure 3B**). Then, top 10 highest-ranked hub ISGs, including IFIT1, RSAD2, ISG15, IFIT3, MX1, USP18, IFI44, OAS2, IFIT2, and OASL, were identified by “Cytohubba” through the MCC method (**Figure 3C**), and these ISGs had strong correlations with each other.

### The results of RT-qPCR

A total of 58 patients with SLE (7 males and 51 females) and 38 healthy controls (7 males and 31 females) were enrolled in this study. There was no significant difference in age or gender between the two groups (*p* = 0.8177, *p* = 0.3885).

**Figure 4:**
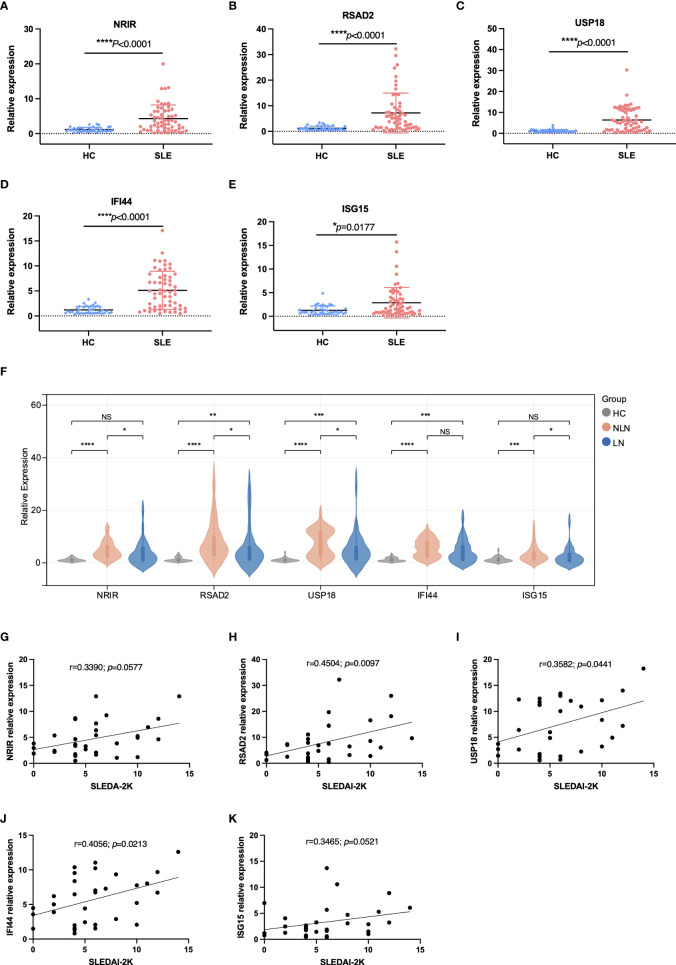
The RT-qPCR results of the validation study for the selected ISGs. **(A-E)** 5 ISGs were significantly differentially expressed between the HC and SLE group. **(F)** 5 ISGs were differentially expressed among the HC, NLN, and LN groups. **(G-K)** The correlations between ISGs relative expressions and SLEDAI-2K scores in the LN group. LN: SLE patients with lupus nephritis; NLN: non-lupus nephritis SLE patients. *P < 0.05, **P < 0.01, ***P < 0.001, ****P < 0.0001, NS no significance (P ≥ 0.05).

The RT-qPCR results showed that lncRNA NRIR and mRNAs (RSAD2, USP18, IFI44, and ISG15) were significantly up-regulated in PBMCs of SLE patients when compared to HCs, (**Figures 4A-E**). NRIR, RSAD2, and USP18 showed stable low expressions in HCs whereas their expression levels varied substantially in SLE patients. All 5 ISGs were up-regulated in more than half of SLE patients.

### Identification of NRIR as a LncRNA closely related to the type I IFN pathway

The expression correlations of 6 lncRNAs and 10 hub ISGs in PBMCs of 20 SLE patients based on GSE122459 were analyzed by Spearman’s test. The results showed that the expressions of NRIR, FAM225A, and LY6E-DT were positively correlated with the expressions of 10 hub ISGs (**Figure 3D**). It’s thought that NRIR, FAM225A, and LY6E-DT were special lncRNAs that can be induced by the stimulation of IFN-α, meanwhile closely related to the Type I IFN pathway.

The results of the Spearman’s test of the relative expression between every two ISGs in the SLE and HC groups separately showed that there were weak to strong positive correlations among 5 ISGs in the HC group (*r*: from 0.3413 to 0.7446), but the positive correlations among these 5 ISGs were more significant and stronger (*r*: from 0.6880 to 0.9177) in the SLE group. The specific correlation coefficients and *p*-values can be found in **Table 3**.

**Table 3:** Correlation coefficients among 5 ISGs in the HC and SLE groups.

		NRIR	RSAD2	USP18	IFI44
HC	RSAD2	0.5810***			
	USP18	0.3413*	0.4921**		
	IFI44	0.6705****	0.7446****	0.5138***	
	ISG15	0.3993*	0.5879***	0.4089*	0.6160****
SLE	RSAD2	0.8287****			
	USP18	0.8310****	0.9177****		
	IFI44	0.8915****	0.9052****	0.9167****	
	ISG15	0.7042****	0.7279****	0.6880****	0.7378****

*p<0.05, **p<0.01, ***p<0.001, ****p<0.0001.

### ISGs are related to LN and disease activity

SLE patients were divided into the lupus nephritis (LN) group and the non-lupus nephritis (NLN) group based on whether or not they had lupus nephritis. There were significant differences in the relative expression of all 5 ISGs between the HC and NLN groups. However, only RSAD2, USP18, and IFI44 expression levels were significantly different between the HC and LN group. In addition, the NLN group had slightly higher levels of NRIR, RSAD2, USP18, and ISG15 expression levels than the LN group (**Figure 4F**).

No significant correlation showed between the relative expression of ISGs and SLEDAI-2K scores in the SLE group. In the LN group, however, there was a significant positive correlation between RSAD2 and USP18 relative expressions and SLEDAI-2K scores (**Figures 4G-K**).

### ISGs are related to clinical symptoms and medication in patients with SLE

All 58 SLE patients were divided into subgroups according to whether or not they displayed additional clinical symptoms. The expression levels of NRIR, USP18, and IFI44 in PBMCs of SLE patients with facial erythema were significantly higher than those in SLE patients without facial erythema (**Table 4**). The expression levels of these 3 ISGs were also higher in Raynaud’s phenomenon positive group than in the negative group. The photoallergic positive group had higher expression levels of NRIR and IFI44 than the negative group. It’s surprising to see that the expression levels of USP18 and IFI44 in SLE patients with pulmonary infection were lower than in SLE patients without pulmonary infection. Pleural effusion was seen in certain SLE patients with pulmonary infection. The expression levels of USP18 and IFI44 were likewise lower in the serositis positive group, which included SLE patients with pleural effusion or pericardial effusion, than in SLE patients without serositis. Thrombocytopenia, one of the typical SLE symptoms, frequently denotes disease activity in SLE patients. We found that SLE patients with thrombocytopenia had considerably lower expression levels of NRIR, RSAD2, and IFI44 than SLE patients with normal platelet counts. Anemia was seen in nearly half of the SLE patients in this study, and the expression levels of RSAD2, USP18, and IFI44 were lower in those patients. More than 15% of the patients in this study had IgG levels that were below the lowest limit of normal value, despite the fact that an increase in IgG level in SLE patients is commonly thought to be clinically linked to disease activity. Notably, the expression levels of 5 ISGs were considerably lower in these patients’ PBMCs.

**Table 4:** The relationship of relative expressions of ISGs in PBMCs with the clinical characteristics of SLE patients.

			NRIR		RSAD2		USP18		IFI44		ISG15	
Characteristics		N	expression	*P*	expression	*P*	expression	*P*	expression	*P*	expression	*P*
**Pulmonary infection**	Yes	15	2.41 (3.84)	0.1747	2.53 (5.25)	0.1664	2.64 (5.03)	**0.0216**	2.44 (3.94)	**0.0406**	0.99 (2.11)	0.1570
	No	43	3.81 (5.31)		5.86 (9.14)		5.98 (9.57)		5.22 (6.58)		1.87 (3.94)	
**Raynaud’s phenomenon**	Yes	5	6.97 (12.78)	**0.0352**	9.64 (15.97)	0.1790	13.44 (18.82)	**0.0168**	8.03 (6.96)	**0.0465**	5.67 (3.52)	0.0710
	No	53	2.95 (4.73)		4.08 (6.86)		4.85 (9.40)		4.42 (5.98)		1.51 (2.69)	
**Photoallergic**	Yes	6	8.64 (10.03)	**0.0151**	8.77 (13.65)	0.0625	9.40 (8.21)	0.0518	8.54 (6.70)	**0.0301**	3.54 (5.27)	0.1526
	No	52	2.99 (4.31)		3.78 (7.01)		4.89 (9.43)		4.38 (6.09)		1.48 (3.00)	
**Facial erythema**	Yes	26	5.33 (6.09)	**0.0016**	5.83 (9.57)	0.1311	6.12 (7.85)	**0.0427**	6.57 (4.88)	**0.0168**	1.81 (4.26)	0.1362
	No	32	1.98 (3.56)		2.96 (6.11)		2.76 (8.84)		2.25 (5.91)		1.31 (3.03)	
**Arthralgia**	Yes	21	2.95 (4.29)	0.9201	6.41 (12.79)	0.2016	6.36 (9.47)	0.5715	5.12 (7.00)	0.5714	2.47 (4.63)	0.7002
	No	37	3.36 (5.56)		3.78 (6.44)		4.92 (9.79)		4.42 (5.70)		1.63 (2.72)	
**Serositis**	Yes	10	1.78 (2.14)	0.0673	1.79 (5.15)	0.0583	1.25 (5.28)	**0.0165**	2.03 (2.99)	**0.0176**	0.71 (2.37)	0.0618
	No	48	3.82 (5.17)		5.66 (8.79)		5.65 (9.44)		5.17 (6.51)		1.81 (3.64)	
**Laboratory test**												
**WBC↓**	Yes	9	6.97 (8.09)	**0.0114**	6.06 (19.02)	0.0898	5.57 (7.70)	0.0658	7.28 (6.07)	0.1002	3.26 (4.49)	**0.0270**
	No	42	2.24 (3.86)		2.96 (6.36)		2.98 (8.98)		3.23 (5.90)		1.03 (2.54)	
**PLT↓**	Yes	7	0.88 (3.06)	**0.0420**	1.36 (2.13)	**0.0249**	2.33 (4.19)	0.0812	1.23 (1.67)	**0.0118**	0.83 (0.89)	0.2181
	No	44	3.50 (5.08)		4.64 (8.13)		5.03 (9.68)		4.45 (6.76)		1.64 (2.99)	
**Anemia**	Yes	26	1.99 (5.78)	0.1895	1.68 (5.75)	**0.0277**	3.54 (5.35)	**0.0189**	2.25 (5.78)	**0.0118**	1.02 (2.57)	0.1212
	No	23	3.71 (5.68)		5.86 (8.17)		5.13 (10.21)		6.47 (7.62)		1.87 (3.83)	
**ESR↑**	Yes	32	2.57 (5.64)	0.5198	3.47 (7.91)	0.6496	4.84 (8.64)	0.2787	4.14 (6.23)	0.5589	1.58 (3.09)	0.8227
	No	14	3.72 (6.18)		3.93 (8.39)		5.00 (9.69)		4.75 (6.91)		1.23 (2.48)	
**CRP↑**	Yes	25	2.69 (4.05)	0.5978	3.39 (9.41)	0.6558	3.73 (7.89)	0.3440	3.85 (5.98)	0.5641	1.63 (3.09)	0.7936
	No	31	3.63 (5.31)		5.13 (6.66)		5.57 (9.06)		5.01 (6.53)		1.65 (3.21)	
**IgG↓**	Yes	8	0.81 (1.45)	**0.0006**	1.33 (4.59)	**0.0274**	1.52 (5.12)	**0.0331**	1.43 (3.08)	**0.0248**	0.71 (0.60)	**0.0216**
	No	42	4.52 (5.11)		5.76 (9.13)		5.85 (10.09)		5.53 (6.07)		1.81 (2.98)	
**IgA↑**	Yes	22	4.78 (4.86)	**0.0259**	6.13 (10.04)	0.2113	5.95 (8.40)	0.1652	5.94 (7.01)	0.0760	2.27 (3.97)	0.2024
	No	28	2.22 (5.31)		3.28 (6.36)		3.74 (9.72)		3.86 (5.63)		1.00 (2.83)	
**C3↓**	Yes	44	3.67 (4.98)	0.5346	5.17 (7.99)	0.6478	5.35 (9.48)	0.5270	5.06 (5.90)	0.5092	1.57 (3.18)	0.1995
	No	6	1.73 (9.19)		4.14 (10.19)		3.57 (11.01)		2.66 (9.88)		1.16 (1.93)	
**C4↓**	Yes	29	3.84 (4.66)	0.7739	6.19 (8.76)	0.5359	6.27 (9.90)	0.1947	5.22 (6.99)	0.2233	1.63 (3.10)	0.6682
	No	21	2.69 (6.89)		2.77 (6.03)		3.22 (7.96)		2.44 (5.97)		1.47 (2.85)	
**HCQ**	Yes	44	2.43 (4.53)	**0.0264**	3.58 (6.19)	**0.0298**	3.99 (8.25)	**0.0227**	3.86 (5.44)	**0.0094**	1.23 (3.00)	0.0732
	No	14	4.78 (4.20)		7.40 (10.28)		8.64 (7.01)		7.86 (5.85)		3.16 (4.15)	
**GC**	Yes	53	2.81 (5.04)	0.1088	3.78 (6.50)	**0.0294**	4.80 (8.15)	**0.0271**	4.10 (5.46)	**0**.**0181**	1.48 (3.31)	0.2306
	No	5	5.50 (4.62)		14.37 (14.46)		11.95 (3.89)		9.51 (3.05)		3.16 (4.20)	

Relative expressions of ISGs are shown as the Median (IQR). WBC, white blood cell; ESR, erythrocyte sedimentation rate; CRP, c-reactive protein; IgG/A, immunoglobulin G/A; C3/C4, complement 3/4; HCQ, hydroxychloroquine; GC, glucocorticoid. Statistically significant *P*-values are in bold.

↑, above the upper limit of the normal value; ↓, below the lower limit of the normal value.

**Figure 5:**
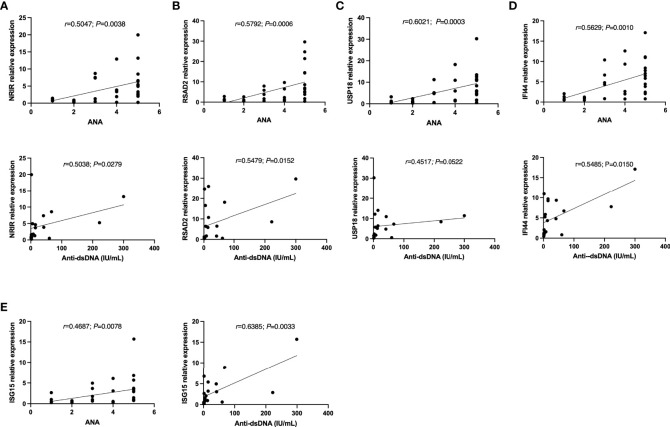
The correlations between **(A)** NRIR, **(B)** RSAD2, **(C)** USP18, **(D)** IFI44, **(E)** ISG15 relative expression and Autoantibodies levels. Note: ANA: antinuclear antibodies; Anti-dsDNA: anti-double-strand DNA antibody. ANA=1: ANA<55 U/mL; ANA=2: 55 U/mL≤ANA-d<100 U/mL; ANA-d=3: 100 U/mL≤ANA-d<200 U/mL; ANA-d=4: 200 U/mL≤ANA-d ≤ 500 U/mL; ANA-d=5: ANA>500 U/mL.

Moreover, these ISGs appeared to be more likely to display higher expression levels in autoantibodies positive SLE patients. As **Table 5** listed, the expression levels of USP18 and IFI44 were higher in the antinuclear antibodies (ANA) positive group than in the negative group. The antinuclear chromatin antibody positive group had higher levels of NRIR and USP18 expression than the negative group. Similarly, in the anti-Smith antibody positive group, NRIR, IFI44, and ISG15 were expressed more highly than in the negative group. Furthermore, we tried to analyze the correlation between the expression levels of ISGs and ANA quantification or anti-dsDNA levels. Results as shown in **Figure 5**, the expression levels of 5 ISGs were positively correlated with the ANA quantification, and the expression levels of all ISGs except USP18 were positively correlated with the anti-dsDNA levels.

**Table 5:** The relationships of relative expressions of ISGs in PBMCs with the autoantibodies of SLE patients.

			NRIR		RSAD2		USP18		IFI44		ISG15	
Autoantibodies		N	expression	*P*	expression	*P*	expression	*P*	expression	*P*	expression	*P*
**ANA**	+	27	3.36 (6.48)	0.0939	4.76 (7.30)	0.1522	5.13 (9.57)	**0.0274**	4.42 (7.14)	**0.0278**	1.04 (3.04)	0.3158
	−	4	0.98 (0.67)		1.26 (1.59)		0.93 (2.24)		1.02 (1.33)		0.79 (1.91)	
**Anti-dsDNA**	+	26	4.12 (4.92)	0.4120	6.45 (13.17)	0.0736	6.12 (7.04)	0.1930	5.53 (5.89)	0.1828	2.07 (2.69)	0.1164
	−	23	2.68 (6.13)		2.60 (6.73)		2.64 (10.13)		2.44 (6.96)		0.87 (4.67)	
**Anti-Chrom**	+	15	4.93 (5.70)	**0.0131**	6.41 (16.87)	0.0801	5.73 (9.73)	**0.0331**	5.84 (7.80)	0.0659	2.09 (4.11)	0.1417
	−	7	0.88 (0.94)		1.04 (5.37)		0.75 (4.59)		1.23 (3.51)		0.72 (2.37)	
**Anti-SSA/Ro52**	+	22	3.60 (6.09)	0.8700	4.94 (5.71)	0.4688	5.03 (9.41)	0.3884	4.94 (5.74)	0.9032	1.55 (4.43)	0.8245
	−	25	3.81 (4.89)		6.19 (13.10)		5.98 (9.62)		5.01 (7.36)		1.76 (2.72)	
**Anti-SSA/Ro60**	+	29	4.63 (6.08)	0.3419	5.20 (13.78)	0.4979	5.13 (9.27)	0.5329	5.22 (6.27)	0.5545	1.87 (4.22)	0.2917
	−	18	3.16 (3.69)		5.14 (7.79)		5.83 (7.80)		4.15 (6.30)		1.23 (3.27)	
**Anti-SSB**	+	10	5.81 (6.19)	0.2131	5.63 (11.16)	0.7445	6.06 (7.97)	0.6959	6.59 (5.99)	0.3921	2.76 (4.21)	0.5643
	−	37	3.36 (5.08)		5.13 (8.91)		5.57 (9.32)		4.48 (6.06)		1.51 (3.70)	
**Anti-Sm**	+	28	4.64 (5.13)	**0.0093**	6.13 (8.86)	0.0727	5.95 (7.29)	0.0855	6.25 (8.18)	**0.0207**	2.76 (4.27)	**0.0210**
	−	20	1.92 (4.59)		3.16 (9.55)		2.68 (9.85)		3.00 (6.04)		0.90 (1.90)	
**Anti-C1q**	+	8	5.80 (4.78)	0.2026	7.53 (18.27)	0.2333	9.59 (6.22)	0.1209	8.06 (7.53)	**0.0373**	2.99 (3.75)	0.1325
	−	23	2.03 (4.66)		2.77 (5.37)		3.22 (4.80)		2.06 (5.58)		0.97 (2.70)	
**Anti-Centromere**	+	6	4.14 (2.67)	0.6561	5.60 (4.29)	0.6932	5.33 (5.07)	0.8689	5.48 (4.96)	0.9352	3.33 (4.53)	0.6519
	−	41	3.81 (6.09)		5.20 (9.62)		5.57 (9.52)		4.77 (7.13)		1.65 (2.88)	
**Anti-RNPA**	+	11	4.63 (4.84)	0.3686	5.46 (24.12)	0.4679	5.57 (10.80)	0.2832	5.84 (8.13)	0.2066	2.09 (6.41)	0.7762
	−	13	1.42 (4.38)		1.64 (8.82)		4.75 (7.14)		4.33 (6.31)		1.51 (3.33)	
**Anti-nucleosome**	+	20	2.99 (5.73)	0.8922	5.17 (7.00)	0.9433	5.43 (7.68)	0.9744	4.60 (5.78)	0.9745	1.54 (3.80)	0.4007
	−	21	3.36 (7.03)		3.78 (6.94)		4.92 (10.95)		4.33 (8.60)		1.02 (2.79)	
**Anti-histone**	+	12	3.55 (5.25)	0.3705	5.63 (5.15)	0.7983	5.95 (5.01)	0.5150	5.06 (4.23)	0.6505	2.44 (3.91)	0.6505
	−	11	5.43 (5.75)		4.76 (11.89)		11.23 (10.71)		6.47 (7.94)		1.45 (4.91)	

Relative expressions of ISGs are shown as the Median (IQR). ANA, antinuclear antibodies; Anti-dsDNA, anti-double-strand DNA antibody; Anti-Chrom, anti-nuclear chromatin antibody; Anti-SSA/B, anti-Sjogren syndrome A/B antibody; Anti-Sm, anti-Smith antibody; Anti-C1q, anti-Complement 1q antibody; Anti-RNPA, anti-ribonucleoprotein antibody A. Statistically significant *P*-values are in bold.

Most of the SLE patients in this study used HCQ and GC regularly to control the disease. The expression levels of NRIR, RSAD2, USP18, and IFI44 in the HCQ-treated group were significantly lower than those in the HCQ-untreated group. Similarly, the expression levels of RSAD2, USP18, and IFI44 in the GC-treated group were significantly lower than those in the GC-untreated group.

### ISGs are related to laboratory test parameters of patients with SLE

We gathered as much laboratory data on SLE patients as possible. By analyzing the correlations between the relative expressions of ISGs and laboratory index levels in SLE patients, it was found that the expression levels of 5 ISGs were significantly negatively correlated with the counts of white blood cells, neutrophils, monocytes, and reticulocytes. The expression levels of NRIR, USP18, and IFI44 were positively correlated with the proportion of lymphocytes. And the expression level of USP18 was negatively correlated with the erythrocyte sedimentation rate (ESR). Furthermore, the ISG15 expression level was positively correlated with the albumin level. The expression level of NRIR was positively correlated with serum IgA level (**Table 6**).

**Table 6:** The correlations between the relative expressions of ISGs and laboratory test parameters.

Laboratory test parameters	Case	NRIR		RSAD2		USP18		IFI44		ISG15		
		*r*	*P*	*r*	*P*	*r*	*P*	*r*	*P*	*r*	*P*
WBC	51	-0.5195	**<0.0001**	-0.4250	**0.0019**	-0.4479	**0.0010**	-0.4448	**0.0011**	-0.5253	**<0.0001**
NEU	51	-0.4624	**0.0006**	-0.4057	**0.0031**	-0.4712	**0.0005**	-0.4107	**0.0028**	-0.4291	**0.0017**
LYM	51	-0.0454	0.7519	-0.0956	0.5045	0.0203	0.8876	-0.0246	0.8640	-0.2371	0.0939
MON	51	-0.3156	**0.0241**	-0.3148	**0.0244**	-0.2955	**0.0353**	-0.2853	**0.0424**	-0.3750	**0.0067**
LYM%	51	0.2971	**0.0343**	0.2643	0.0609	0.3789	**0.0061**	0.2866	**0.0415**	0.1585	0.2667
MON%	51	0.0231	0.8721	0.0037	0.9795	0.0364	0.7996	0.0235	0.8699	0.0314	0.8270
PLT	51	0.2173	0.1256	0.2659	0.0593	0.1598	0.2628	0.2597	0.0658	0.2036	0.1519
RC	50	-0.3654	**0.0091**	-0.3532	**0.0119**	-0.4765	**0.0005**	-0.3989	**0.0041**	-0.3002	**0.0342**
ESR	46	-0.1900	0.2059	-0.2042	0.1734	-0.3150	**0.0330**	-0.2743	0.0651	-0.1746	0.2457
Hb	49	0.1780	0.2211	0.2897	**0.0435**	0.3057	0.0326	0.3181	**0.0259**	0.2197	0.1293
CRP	56	-0.0758	0.5789	0.0575	0.6738	-0.1546	0.2551	-0.1117	0.4125	-0.0823	0.5467
Alb	55	0.2251	0.0985	0.1525	0.2662	0.1491	0.2772	0.2106	0.1228	0.2877	**0.0332**
IgG	50	0.2625	0.0656	0.07328	0.6130	0.1237	0.3919	0.1831	0.2031	0.1562	0.2787
IgA	50	0.3183	**0.0243**	0.1571	0.2760	0.1864	0.1950	0.2279	0.1114	0.1835	0.2021
IgM	50	0.2171	0.1299	0.1594	0.2687	0.1834	0.2023	0.1335	0.3555	0.1954	0.1738
C3	50	-0.0405	0.7801	0.0108	0.9409	-0.0571	0.6937	-0.0990	0.4941	-0.0470	0.7458
C4	50	-0.0542	0.7085	-0.1374	0.3413	-0.1844	0.1998	-0.1789	0.2139	-0.0854	0.5553

NEU, neutrophils; LYM, lymphocyte; MON, monocyte; PLT, platelet; RC, reticulocyte; Hb, Hemoglobin; Alb, albumin. Statistically significant *P*-values are in bold.

### IFI44, USP18, and RSAD2 have a good diagnostic efficacy for SLE.

**Figure 6:**
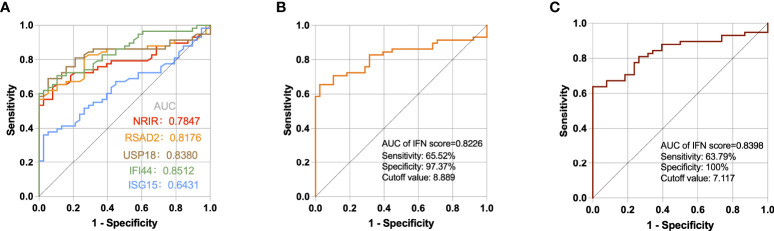
ROC curves for the **5** ISGs **(A)**, IFN score calculated by 5 ISGs **(B)**, and IFN score calculated by IFI44, USP18, and RSAD2 **(C)** in SLE patients compared with healthy controls.

Through ROC curve analysis, we evaluated if a single ISG expression level and IFN score may assist differentiate SLE patients from healthy people. The areas under the curves (AUCs) of NRIR, RSAD2, USP18, IFI44, and ISG15 were 0.7847, 0.8176, 0.8380, 0.8512, and 0.6431 respectively (**Figure 6A**). According to a guide to evaluating the utility of biomarkers based on AUC (28), IFI44, USP18, and RSAD2 can all be used as good indicators for differentiating SLE patients from healthy controls while NRIR was considered fair, but ISG15 had a poor capability. In addition, the IFN score calculated by 5 ISGs also performed well (AUC=0.8226), and when the cutoff value of the IFN score was 8.889, the sensitivity and specificity for the diagnosis of SLE were 65.52% and 97.37% (**Figure 6B**). The three ISGs (IFI44, USP18, and RSAD2) with the best diagnostic performance were used to calculate a second IFN score, with a higher AUC value of 0.8398 (**Figure 6C**). It seems like a single gene level appears to be easier to be detected, but the IFN score is more stable.

## Discussion

SLE is a classic autoimmune disease, with an annual incidence ranging from 1.5 to 11 per 100,000 people (29). The mortality rate of SLE is two to three times higher than the general population, with young women bearing the brunt of the burden (29). As a result, a more comprehensive and in-depth understanding of the mechanism is urgently needed in order to create new, sound strategies for diagnosing and treating SLE. At present, the diagnosis of SLE depends primarily on clinical manifestations and laboratory indicators like autoantibodies levels. To improve the sensitivity and specificity of SLE diagnosis, researchers are now continuously working on novel biomarkers. Therefore, the future of precision therapy may be to deliver individualized treatment after molecular stratification.

Type I IFN system has been the focus of SLE-related research for a long time. High levels of IFN signature can be found in 60% to 80% of SLE patients, as additionally proven by the high-throughput sequencing and gene chip technology (16, 30). Although the type I IFN system is also active in rheumatoid arthritis, primary Sjogren’s syndrome, and other autoimmune disorders (31, 32), its intensity is much lower than that in SLE (30, 32). The researchers found that the IFN signature recognized in PBMCs of SLE patients seemed to be more sensitive to the activation of the IFN pathway than serum IFN-α levels (33), that’s maybe why most studies focused on detecting the transcriptional levels of ISGs in peripheral blood to find candidate biomarkers for diagnosis and treatment of SLE patients.

Our study first screened the DEISGs, including lncRNAs and mRNAs, in PBMCs of SLE patients and healthy individuals by analyzing datasets files. GO enrichment analysis showed that these differentially expressed mRNAs were mainly engaged in response to virus, immune response, chemokine activation, and RNA binding, implying that ISGs might participate in the onset and progression of SLE through these biological processes. The KEGG analysis revealed that these differentially expressed mRNAs were mostly concentrated in virus infection-related diseases, suggesting that the functions of these ISGs in SLE might be similar to those in these diseases, which helped to explore the mechanisms of SLE. The GO and KEGG results point us in the right path for further research into how these genes work.

The next phase of our study was to identify hub ISGs in SLE for indicating subgroups of SLE patients with certain clinical characteristics using fewer observational markers. Thus, we identified hub ISGs in the PPI network and found that there were strong interactions among IFIT1, RSAD2, IFIT3, USP18, ISG15, OASL, MX1, OAS2, OAS3, and IFI44, all of which are meanwhile potential biomarkers closely involved in SLE. Earlier reports have reported these ISGs (30, 34). IFIT1 and IFIT3 belong to the IFN-induced protein with the tetratricopeptide repeats (IFIT) family (35). IFIT3 expression in human diploid fibroblasts cells activates TBK1 and IRF3, causing the production of IFN-β, activation of STAT1, and induction of ISGs expression (35). RSAD2, also known as Viperin, is a highly induced ISG and plays an intermediary role in inducing pDCs to generate IFN-I by mediating TLR7 and TLR9 (36). Silencing RASD2 can reduce the viability and promote the apoptosis of CD19^+^ B cells by inhibiting NF-κB pathway, as well as reduce the expression of IL-10 (37). RSAD2 has been identified as a key gene of SLE frequently, not only its transcriptional levels but also serum protein levels were significantly up-regulated in SLE patients (34). USP18 and ISG15 are 2 ISGs that are mutually exclusive (38). ISG15 is one of the most strongly induced type I ISGs and causes the ISGylation of hundreds of host and viral proteins by covalently binding to the target protein, whereas USP18 can specifically remove ISG15 from the protein conjugate of ISGylation and further hydrolyze ISG15 (39). In addition to DeISGylation, USP18 functions as a negative regulator of the IFN response by inhibiting the JAK/STAT pathway (40). The interaction between ISG15 and USP18 maintains the balance of the IFN system (41). OAS2, OAS3, and OASL all belong to the 2′-5′-oligoadenylate synthetases (OAS) family, which are the only proteins known to catalyze 2’-specific nucleotidyl transfer, induced by IFN and activated by dsRNA (42). These ISGs not only play a direct antiviral role but also maintain the balance of the type I IFN system through positive or negative regulation. Imbalanced type I IFN signaling results in immune system disorder, one of the causes of chronic inflammation and autoimmune diseases such as SLE (43).

Among the 6 lncRNAs screened upregulating in PBMCs of SLE patients, we found that NRIR, FAM225A, and LY6E-DT were special potential ISGs and positively correlated with a part of ISGs. According to genome position, NRIR is adjacent with ISGs (CMPK2 and RSAD2), and LY6E-DT neighbors ISG LY6E, which has previously been considered a good biomarker for SLE diagnosis previously (30). LncRNAs can regulate the expression of co-expressed and co-located mRNAs through *trans*-acting and *cis*-acting (44). So, these 3 lncRNAs could have regulatory effects on IFN signature. Among them, NRIR, also known as lncCMPK2, has been proved to be a bona fide ISG induced by IFN-I *via* JAK/STAT pathway (45, 46). Intriguingly, the regulatory effect of NRIR seems to be different in different types of cells. Researchers Kambara et al. found that NRIR knockdown resulted in the upregulation of several protein-coding antiviral ISGs in hepatocytes (45). However, in monocytes, NRIR knockdown mainly reduced the LPS-induced expression of IFN-I target genes suggesting that NRIR is a positive regulator of ISGs according to researchers Mariotti et al. (46). FAM225A was found to be up-regulated in some tumors such as nasopharyngeal carcinoma and gastric cancer and to promote tumorigenesis and development *via* sponging miRNAs (47, 48). LY6E-DT was identified to be down-regulated in hepatocellular carcinoma and high-grade serous ovarian cancer (49, 50). However, there are few publications on the mechanism of LY6E-DT, and we guess that its proximity to LY6E may be a good start in exploring potential functions.

After that, we combined the RT-qPCR results and clinical data to clarify the ability of 5 ISGs (NRIR, RSAD2, USP18, IFI44, and ISG15) as biomarkers for diagnosis and stratification of SLE. These 5 ISGs in PBMCs were significantly up-regulated in more than half of the SLE patients, consistent with the results of bioinformatics analysis. Meanwhile, there was a stronger positive correlation among 5 ISGs in SLE patients than in healthy controls, demonstrating the overactivation of the IFN-I system in most SLE patients. We did not detect direct correlations between ISGs expression levels and SLEDAI-2K score, as several longitudinal investigations of SLE reported that the IFN signature in patients with SLE maintained a relatively stable level and did not change over time (3, 16, 51). In LN patients from our study, on the other hand, the expression levels of RSAD2, USP18, and IFI44 were significantly positively with the SLEDAI-2K score. Previous studies have reported that IFI44 levels in active LN patients are significantly higher than in inactive LN patients, which is consistent with part of our results (52). Thus, some ISGs may play a key role in the development of lupus nephritis. We hypothesize that the expression levels of some ISGs could be performed as a long-term rather than short-term indicator of SLE since lupus nephritis often indicates a more persistent and severe disease in SLE patients.

By analyzing the correlation between 5 ISGs expression levels and basic or clinical characters, we found that there was a negative correlation between expression levels of ISGs (RSAD2, USP18, IFI44, and ISG15) and the age of SLE patients, while this correlation was not observed in HCs, implying that higher IFN signature might be associated with the pathogenesis of younger SLE patients. But there are few reports about the relationship of age and IFN signature in SLE. In terms of clinical symptoms, we discovered that patients with high expression of most ISGs were more likely than patients with low expression to suffer rash-related symptoms, which is consistent with previous research (53). SLE is induced or aggravated to some extent by factors including UV, hormones, and infection (10), some of which may role as triggers of the IFN system (54). Raynaud’s phenomenon refers to paroxysmal spasm of peripheral arterioles, which is common in autoimmune diseases. It has been reported that patients with tumor develop Raynaud syndrome after IFN- α treatment (55), suggesting that the IFN system may have a direct peripheral vascular toxicity of SLE patients. As for laboratory data, we found that the expression levels of 5 ISGs were strongly adversely linked with the counts of various blood cells such as white blood cells, neutrophils, and monocytes, as previously reported by other studies (56). Both innate and adaptive immunological changes that affect blood cell composition are seen in SLE patients (57). Abnormal immune cell death and clearance disorders may lead to the collapse of autoimmune tolerance. For example, neutrophil extracellular traps (NETs) are special way of neutrophil death, which may have different effects on immune response and tissue damage in the context of autoimmunity. NETs activate pDCs to produce IFN-I while IFN-I can promote the formation of NETs in the case of neutrophil imbalance. The interaction between them leads to a vicious circle of persistent inflammation (57, 58). Moreover, researchers found that some ISGs like IFIT4 might play roles in promoting monocyte differentiation into dendritic cell-like cells (59). The above reports may explain partly the negative correlation between partial blood cell count and ISG expression in patients with SLE in this study. Obviously, SLE patients with higher IFN signature show cytopenia more possibly, suggesting that some ISG expression might signal blood system damage in SLE patients.

SLE is currently diagnosed mainly through the detection of autoantibodies. Our study analyzed the correlation of levels between target ISGs and certain autoantibodies. SLE patients with positive ANA, anti-Chrom, anti-Sm or anti-C1q were more likely to show ISGs overexpression according to the results. Furthermore, ISG expression levels were positively correlated with ANA and anti-dsDNA titer, the latter of which is considered an autoantibody indicator for renal involvement and activity. It is reported that the levels of IFN-I and some autoantibodies increased in patients with SLE a few years before the onset of the disease (60). In the years before the diagnosis of SLE, some autoantibodies increased to the diagnostic critical level, and these autoantibodies formed nucleic acid immune complexes to induce IFN-I production (60). But it is unclear whether some ISGs contribute to the formation of autoantibodies like anti-dsDNA antibodies, which consequently worsen the condition of SLE patients. Therefore, it remains to be clarified whether the two contribute to one another. Finally, ROC curve analysis revealed that IFI44, USP18, RSAD2, and NRIR had good diagnostic efficacy for identifying SLE patients. In this study cohort, the positive rate of anti-dsDNA in SLE patients was 53.06%. Compared with anti-dsDNA, IFN signature can help identify more SLE patients but may sacrifice specificity. Combining IFN signature or several ISGs as new biomarkers with existing diagnostic indicators may greatly improve the efficiency of clinical diagnosis.

We also found that expression levels of ISGs in PBMCs of SLE groups with HCQ or GC treated were lower than in groups without treatment of HCQ or GC, suggesting that HCQ and GC have a certain inhibitory influence on IFN signature. Studies have reported that conventional doses of GC hardly affect the IFN signature while large doses of GC can temporarily block the expression of type I ISGs by depleting the pDCs in the blood (16, 61). HCQ is a safe and efficient treatment for SLE. Studies have tentatively demonstrated that HCQ can inhibit IFN-α production of pDCs and expression of ISGs in SLE patients (62, 63).

Even while these findings are promising, there has to be a lot more investigation into the specifics of the mechanisms. We think the IFN system has a complicated, potentially pathogenic, and protective function in SLE. The interpretation of SLE is greatly aided by an understanding of the IFN system balance. Among the ISGs, we were particularly interested in NRIR, a member of the lncRNA family that may regulate the expression of protein-coding genes and a variety of biological processes. In the near future, we’ll work to clarify the mechanisms of NRIR in the IFN pathway and the immunological response to SLE. Furthermore, we cannot ignore the limitations of this study. To begin with, we enrolled SLE patients with lenient criteria in order to look at as many possible connections between ISGs and SLE. However, the promotion of IFN signature may be influenced by a multitude of factors in either a synergistic or antagonistic role. To more accurately depict the correlations between ISGs and each variable, we need to either perform longitudinal research or increase the sample size based on stringent case inclusion criteria later. Secondly, further research is needed to determine if these key ISGs can distinguish SLE patients from patients with other autoimmune disorders. Thirdly, we chose PBMC due to its accessibility in a clinical environment. The cell composition of SLE patients’ peripheral blood differs greatly, though, and we think that distinct cell subsets contribute to IFN signature in different ways. Separating distinct cell subsets for investigating the activities of IFN signature could therefore be valuable for enriching pathogeny and developing targeted therapy for SLE patients.

## Conclusion

In conclusion, our study identified a few ISGs as potential diagnostic biomarkers of SLE by bioinformatics tools and discovered a series of pathologies in SLE that might be affected by type I ISGs by combining RT-qPCR results and clinical data. Our findings contribute to confirming several reliable ISGs for the delectation of IFN system status in SLE patients and provide ideas for diagnosis and treatment targets as well as etiological mechanisms of SLE.

## Data availability statement

The original contributions presented in the study are included in the article/**supplementary material**. Further inquiries can be directed to the corresponding author.

## Ethics statement

The studies involving human participants were reviewed and approved by Human Ethics Committee of Bengbu Medical College. Written informed consent to participate in this study was provided by the participants’ legal guardian/next of kin.

## Author contributions

TW and MS presented the idea and design. MS analyzed and interpreted data. MS and CD collected samples, performed experiments, and wrote the manuscript. CX and HW reviewed the conclusions and revised the manuscript. ZL and BL supervised the whole study. All authors contributed to the article and approved the submitted version.

## Funding

This work was supported by the Natural Science Foundation of Anhui Provincial (2108085MH258).

## Acknowledgments

We thank Maria Tokuyama, Jamie Gearing, et al. for uploading the expression profiling of high throughput sequencing. We would like to thank every author who completed this work and the academic editor and reviewers for improving the quality of this article. Finally, we are grateful to all of the patients who participated in this study.

## Conflict of interest

The authors declare that the research was conducted in the absence of any commercial or financial relationships that could be construed as a potential conflict of interest.

## Publisher’s note

All claims expressed in this article are solely those of the authors and do not necessarily represent those of their affiliated organizations, or those of the publisher, the editors and the reviewers. Any product that may be evaluated in this article, or claim that may be made by its manufacturer, is not guaranteed or endorsed by the publisher.

## Footnotes

1 Gene Expression Omnibus (2021) (http://www.ncbi.nlm.nih.gov/geo/).

2 Sangerbox (2021) (http://sangerbox.com/).

3 Bioinformatics & Evolutionary Genomics (http://bioinformatics.psb.ugent.be/webtools/Venn/).

4 ClustVis (2015) (https://biit.cs.ut.ee/clustvis/).

5 GeneCodis (2021) (https://genecodis.genyo.es).

6 STRING (2022) (https://cn.string-db.org).
