# Supplementary material for: Identification of key interferon-stimulated genes for indicating the condition of patients with systemic lupus erythematosus
Source: Front Immunol. 2022 Jul 28;13:962393. doi: 10.3389/fimmu.2022.962393 (PMC9365928; doi:10.3389/fimmu.2022.962393)
Supplement: Supplementary file 1 [file Table_1.docx]

Supplementary Material

**Supplementary Table 1 |** A brief introduction to datasets

| GEO accession | Platforms | Country | Groups | Cell Type |
| --- | --- | --- | --- | --- |
| GSE122459 | GPL16791;  GPL18573 | USA | SLE=20;  HC=6 | PBMC |
| GSE159094 | GPL18573 | Australia | IFN-α 2a treated=4;  Untreated=4 | PBMC |

**Supplementary Table 2 |** Primer sequences of 5 ISGs and 1 internal reference gene

| Primer | Sequence（5’🡪3’） |
| --- | --- |
| β-actin | F：CACGAAACTACCTTCAACTCC  R：CATACTCCTGCTTGCTGAT |
| NRIR | F：CCTTGGCAACTGCTCACGAT  R：AGGAGGTTAGAGGTGTCTGCT |
| RSAD2 | F：CAGCGTCAACTATCACTTCACT  R：AACTCTACTTTGCAGAACCTCAC |
| USP18 | F：CCTGAGGCAAATCTGTCAGTC  R：CGAACACCTGAATCAAGGAGTTA |
| IFI44 | F：ATGGCAGTGACAACTCGTTTG  R：TCCTGGTAACTCTCTTCTGCATA |
| ISG15 | F：CGCAGATCACCCAGAAGATCG  R：TTCGTCGCATTTGTCCACCA |

**Supplementary Table 3 |** Correlation between age and relative expression level of ISG in SLE group and HC group

|  | Variable | *P* value | *r* value |
| --- | --- | --- | --- |
| NRIR in SLE | Age | 0.1494 | -0.1917 |
| NRIR in HC |  | 0.9662 | 0.007117 |
| RSAD2 in SLE |  | **0.0219^*^** | -0.3006 |
| RSAD2 in HC |  | 0.5561 | 0.09855 |
| USP18 in SLE |  | **0.0176^*^** | -0.3107 |
| USP18 in HC |  | 0.3609 | -0.1524 |
| IFI44 in SLE |  | **0.0075^**^** | -0.3479 |
| IFI44 in HC |  | 0.9982 | -0.0003833 |
| ISG15 in SLE |  | **0.0265^*^** | -0.2913 |
| ISG15 in HC |  | 0.7834 | -0.04610 |
